# An Upstream Hfq Binding Site in the *fhlA* mRNA Leader Region Facilitates the OxyS-*fhlA* Interaction

**DOI:** 10.1371/journal.pone.0013028

**Published:** 2010-09-28

**Authors:** Nilshad N. Salim, Andrew L. Feig

**Affiliations:** Department of Chemistry, Wayne State University, Detroit, Michigan, United States of America; Centre de Regulació Genòmica, Spain

## Abstract

**Background:**

To survive, bacteria must be able to adapt to environmental stresses. Small regulatory RNAs have been implicated as intermediates in a variety of stress-response pathways allowing dynamic gene regulation. The RNA binding protein Hfq facilitates this process in many cases, helping sRNAs base pair with their target mRNAs and initiate gene regulation. Although Hfq has been identified as a critical component in many RNPs, the manner by which Hfq controls these interactions is not known.

**Methodology/Principal Findings:**

To test the requirement of Hfq in these mRNA-sRNA complexes, the OxyS-*fhlA* system was used as a model. OxyS is induced in response to oxidative stress and down regulates the translation of *fhlA*, a gene encoding a transcriptional activator for formate metabolism. Biophysical characterization of this system previously used a minimal construct of the *fhlA* mRNA which inadvertently removed a critical element within the leader sequence of this mRNA that effected thermodynamics and kinetics for the interaction with Hfq.

**Conclusions/Significance:**

Herein, we report thermodynamic, kinetic and structural mapping studies during binary and ternary complex formation between Hfq, OxyS and *fhlA* mRNA. Hfq binds *fhlA* mRNA using both the proximal and distal surfaces and stimulates association kinetics between the sRNA and mRNA but remains bound to *fhlA* forming a ternary complex. The upstream Hfq binding element within *fhlA* is similar to (ARN)_x_ elements recently identified in other mRNAs regulated by Hfq. This work leads to a kinetic model for the dynamics of these complexes and the regulation of gene expression by bacterial sRNAs.

## Introduction

Small non-coding RNAs (sRNA) mediate gene regulation in both bacteria and eukaryotes [1], [2], [3]. Bacteria commonly employ sRNAs during stress responses, allowing them to survive when exposed to suboptimal growth environments [4]. Two main classes of sRNAs exist in bacteria, *cis* and *trans*-encoded variants. Cis RNAs derive from the same genetic locus as the regulated message but are transcribed from the antisense strand; thus exhibiting perfect complementarity with their target. These RNAs are known to control regulatory pathways such as transcriptional attenuation, RNA processing and decay, and translation initiation [5], [6]. Unlike cis-acting sRNAs, the trans-acting sRNAs are expressed from genetic loci different than their targets and interact using imperfect base pairing. These sRNAs often require accessory proteins such as Hfq for activity.

Hfq is a homohexamer that belongs to the Sm/LSm family of RNA binding proteins [7], [8], [9], [10]. It typically varies in length between 70 and 110 amino acids in *E. coli*, and is highly abundant where an estimated 10,000 hexamers present in the cytoplasmic fraction, often in association with ribosomes [7], [11]. Hfq is mostly conserved among the bacterial kingdom with more than 3000 homologs currently annotated in genomic databases. Mutational studies in *E. coli* and other organisms have shown that strains lacking Hfq exhibit pleiotropic effects such as decreased growth rates, increased stress sensitivity (UV, oxidative and cold shock), ineffective tRNA maturation and mini-cell formation [12], [13], [14]. In addition, it was demonstrated that reduced virulence was observed in the absence of Hfq for a variety of bacterial pathogens [15], [16], [17], [18], [19].

Hfq is known largely for its role in post-transcriptional gene regulation by facilitating pairing between sRNAs and mRNAs. A common feature in these pathways is the presence of overlapping networks of RNA interactions where one sRNA regulates multiple genes. For example the sRNA RybB has been shown to regulate *sodB*, *ftnA*, *bfr*, *acnA* and *sdhC* and thus acts as a regulatory node allowing a complex and integrated response to a given growth condition, in this case low iron concentrations [20]. Although Hfq has been identified as a critical component in these systems, a common mechanism as to how it facilitates complex formation is not clear.

**Figure 1:**
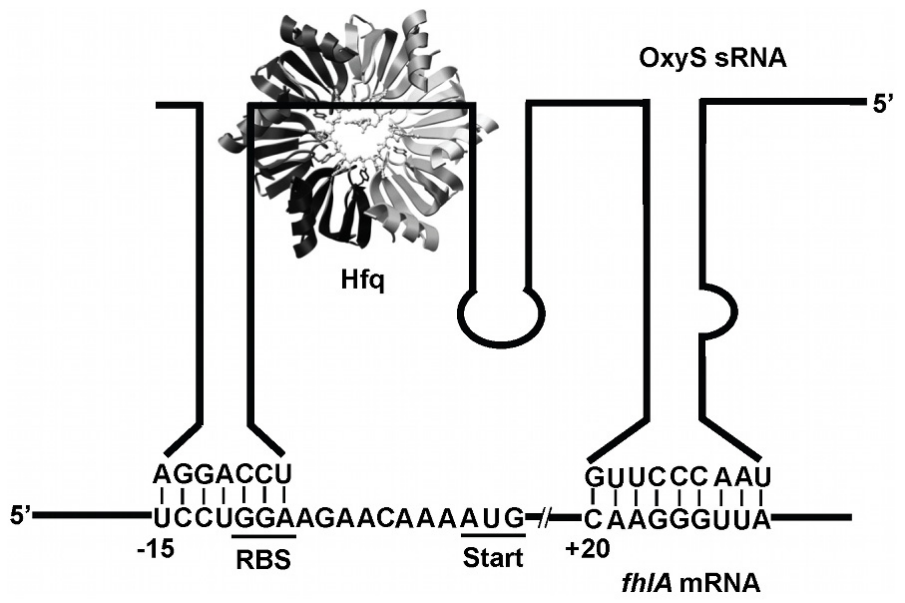
Regulation of *fhlA* by sRNA OxyS in the presence of Hfq. Interaction between *fhlA* mRNA and the sRNA OxyS is shown. *fhlA* encodes a transcription factor for formate metabolism. During oxidative stress the sRNA OxyS is expressed and in the presence of Hfq was proposed to form two kissing interactions [21] through the stem loops present in the mRNA and the sRNA. The interaction formed within the 5′ leader region of *fhlA* sequesters the ribosome binding site preventing translation. The contact within the coding sequence was shown to be important for efficient gene regulation [21].

To further understand the requirement of Hfq during sRNA:mRNA pairing, we have studied the OxyS-*fhlA* system (Fig. 1). OxyS is a regulatory RNA expressed in response to oxidative stress. One of the mRNAs it interacts with is *fhlA*, a message encoding a transcriptional activator for formate metabolism [21]. Interaction at two short pairing elements, one at the RBS and another within the coding region, are sufficient to prevent translation of the *fhlA* mRNA. Previous studies in vivo showed that, in the absence of Hfq, OxyS was unable to regulate the expression of *fhlA* [22]. Most of the work on this regulatory network used a minimal *fhlA* mRNA construct that was sufficient to interact with its sRNA OxyS. A recent study by Soper et al., however, showed a marked difference in the *rpoS*-DsrA interaction with Hfq when the leader sequence of the mRNA was extended. This study showed that Hfq interacted with the *rpoS* mRNA at a novel (ARN)_X_ sequence element [23]. This motif was originally called an AAYAA element by Soper et al. [23] It was subsequently referred to as an (ARE)_x_ element by Link and co-workers who showed based on crystallographic and biochemical studies the site has broader specificity than AAYAA [28]. Unfortunately, the acronym ARE has already been used for many years to refer to A/U-Rich Elements in eukaryotic mRNAs [24], [25]. We therefore propose calling this sequence motif by the name (ARN)_x_ to distinguish it from AREs while still retaining the necessary information about the sequence specificity. To understand whether the (ARN)_x_ element is a commodity among regulatory networks involving Hfq, we tested this hypothesis in the OxyS-*fhlA* system.

Here we show that the extension of the *fhlA* leader sequence enhances the kinetics and thermodynamics of *fhlA* associating to Hfq stimulating ternary complex formation with OxyS. Structural probing of the leader sequence confirmed Hfq-binding to the (ARN)_x_ element at position −76 in the upstream region of the *fhlA* mRNA. Finally, we show that Hfq binds to the *fhlA* leader sequence using both its proximal- and distal-RNA binding surfaces which helps explain the kinetic and thermodynamic properties of Hfq association with mRNAs. These data lead us to revise the model for Hfq-dependent gene regulation to be less sRNA-centric and providing more importance to the manner in which Hfq interacts with a subset of bacterial messages that are subject to regulation by sRNAs.

## Results

### OxyS and Hfq interacts with *fhlA* leader construct to form a ternary complex

**Figure 2:**
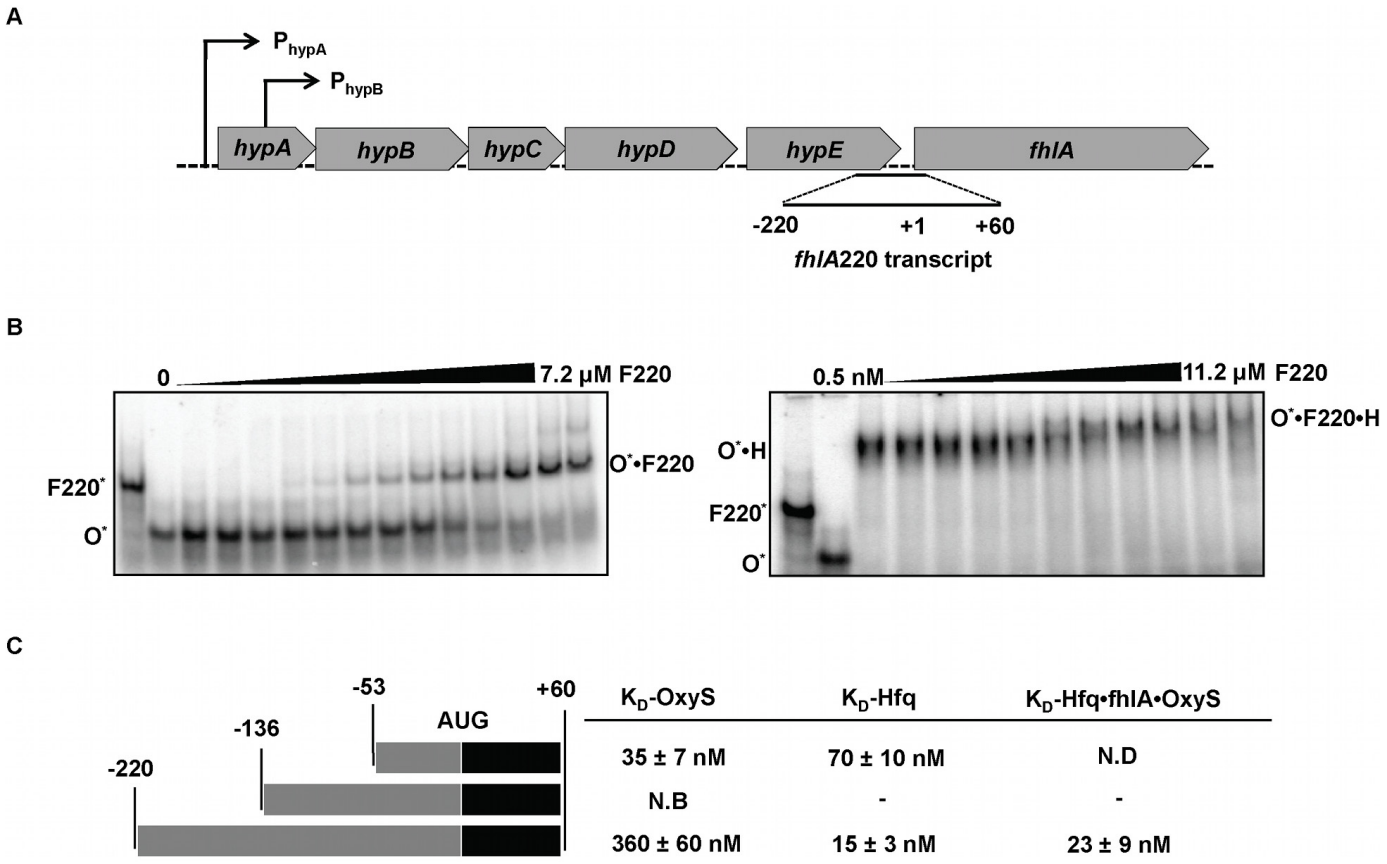
The *fhlA* mRNA leader constructs used in this study. (A) The *fhlA* locus. *fhlA* is transcribed by promoters that belong to the family of hydrogenase iso-enzymes specifically by promoters P_hypA_ and P_hypB_ [24]. (B) Analysis of binary and ternary complexes of *fhlA*220 mRNA with OxyS and Hfq (also see Figure S2). Gel shift experiments showing binary complex formation between OxyS (O^*^) and *fhlA*220 (F220) (left). Uniformly ^32^P-labled OxyS (*) was titrated with varying concentrations of *fhlA*220 ranging from 0.5 nM-7.2 µM. Ternary complex formation between OxyS (O), Hfq (H) and *fhlA*220 (F220) (right). The O•H complex was pre-formed by incubating ∼1 pmol of [5′-^32^P] labeled OxyS with 1 µM Hfq. *fhlA*220 (F220) was titrated from 0.5 nM-11.2 µM. (C) *fhlA* mRNA leader constructs used during this study and their affinities. Three *fhlA* leader constructs were tested. *fhlA*53 was previously characterized by Argaman et al. and comparisons were made relative to this construct. A stable OxyS•*fhlA*136 complex was not detected in gel shift assays due to probable mis-folding of OxyS binding elements. The *fhlA*220 leader sequence showed activity, forming stable binary and ternary complexes with OxyS and Hfq.

To test whether the upstream leader region of *fhlA* facilitates the interaction with OxyS and Hfq as previously described for the *rpoS*-DsrA system [23], the leader sequence of *fhlA* was extended from the previously characterized position −53 relative to the start codon, to −136 and −220 respectively. A putative upstream Hfq-binding element was absent in the minimal *fhlA*53 construct but is encoded at position −76 and thus is present in both extended constructs. RNA transcripts are denoted according to the length of their leader sequence: *fhlA*220 has 220 nt upstream of its start codon. In its genomic context, *fhlA is* part of a polycistronic message, transcribed under the control of two promoters P_hypA_ and P_hypB_ [24] (Fig. 2A).

To test the potential of *fhlA* mRNA leader constructs to undergo post-transcriptional regulation, gel mobility shift assays were used to detect binding to radiolabeled OxyS. Equilibrium experiments were performed with *fhlA*136, but neither binary complex with OxyS nor ternary complexes with OxyS and Hfq were observable using EMSA. For *fhlA*220, on the other hand, both binary and ternary complexes with OxyS and Hfq were stable. The OxyS•*fhlA*220 (O•F220) complex formed with a K_D_ of 360±60 nM (Fig. 2B and Figure S1). This affinity is ∼10-fold less than that of the OxyS•*fhlA53* (O•F53) complex where a K_D_ of 35±7 nM was measured (Fig. 2C). This value compares favorably with the K_D_ previously reported for the O•F53 complex by Argaman et al. in a slightly different binding buffer (10 mM Tris, 60 mM KCl, 10 mM MgCl_2_ and 1 mM DTT (pH 8.0)) [21]. Reduced affinity between OxyS and *fhlA*220 was surprising, but this phenomenon was also observed for DsrA and RprA, both of which bound extended *rpoS* leader sequences 10–20-fold less tightly than to the truncated leader [23], [25].

To detect whether *fhlA*220 interacts with Hfq, [5′-^32^P] *fhlA*220 mRNA was incubated with Hfq at concentrations of 0 nM to 1.67 µM Hfq (hexamer). The Hfq•*fhlA*220 (H•F220) complex was resolved on native gels to provide a K_D_ of 15±3 nM Hfq hexamer (Figure S1 and Figure S2). The F220•H complex formed with ∼5 fold tighter affinity than F53•H (K_D_, 70±10 nM) revealing that the extended structure may present extra Hfq binding elements or a more favorable Hfq-binding motif (Figure S3). The OxyS•*fhlA*220•Hfq ternary complex (O•F220•H) was also resolved using gel shift assays when pre-formed [5′-^32^P] OxyS•Hfq complex was titrated with 0.5 nM–11.2 µM *fhlA*220 mRNA (Fig. 2B), yielding a K_D_ of 23±9 nM. Thus the presence of Hfq provides 15-fold tighter binding between OxyS and *fhlA*, a net stabilization of ∼1.6 kcal/mol.

### *fhlA*220 mRNA interacts with both proximal and distal RNA binding surfaces of Hfq

**Figure 3:**
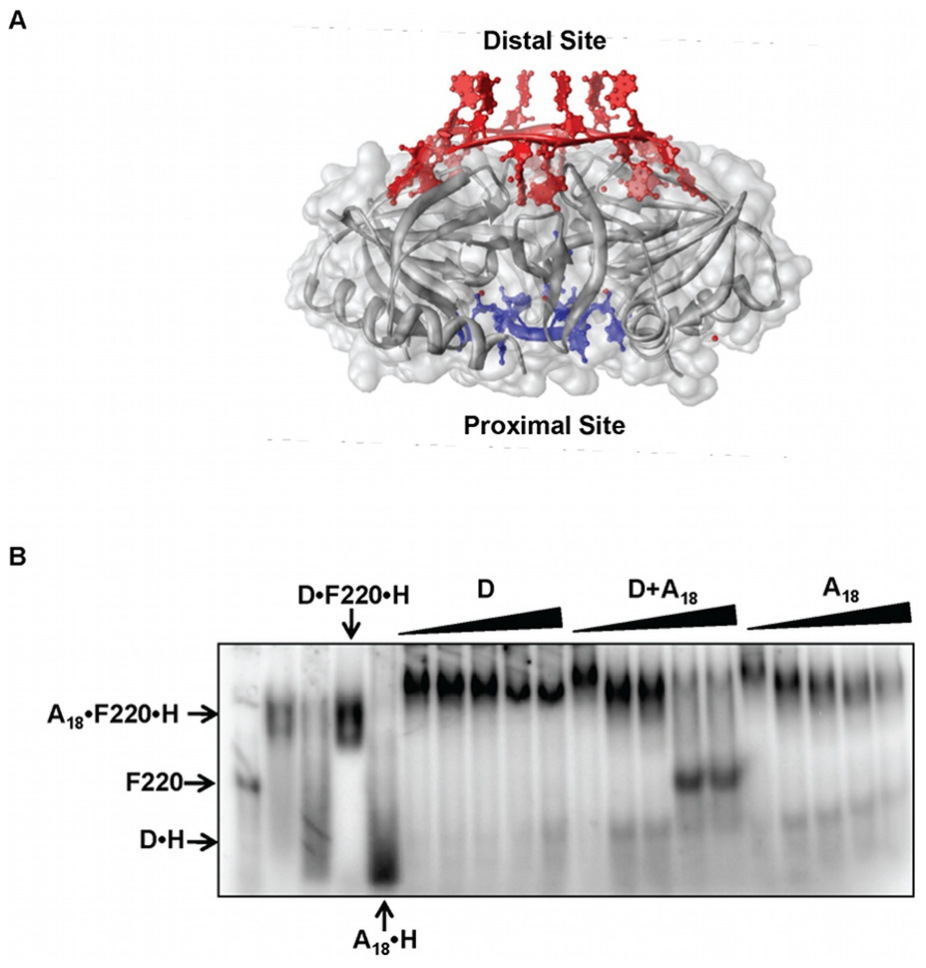
Competition binding experiments to determine the Hfq binding surface that interacts with *fhlA*220. (A) Superposition of two Hfq crystal structures that are crystallized in the presence of (AU_5_G) RNA at the proximal site (1KQ2) and in A_15_ RNA bound at the distal site (3GIB) [10], [26]. Superposition of the two crystal structures were performed using UCSF Chimera software [44]. (B) The complex between [5′-^32^P] *fhlA*220 (F220) and Hfq (H) was pre-formed and incubated with increasing concentrations of A_18_ RNA (0–30 µM), DsrA (0–30 µM) or with both RNAs (0–30 µM). DsrA and A_18_ have been previously shown to specifically bind to proximal and distal RNA binding sites of Hfq respectively [28].

Hfq binds RNAs using two distinct surfaces – a proximal surface that favors U-rich sequences like those in many Hfq-binding sRNAs (such as DsrA and RybB) and the distal surface that binds poly-A RNAs (Fig. 3A) [11], [25], [26], [27]. Competitive binding studies were used to investigate *fhlA*220 mRNA binding to Hfq. [5′-^32^P] H•F220 complex was prepared and then incubated with excess of either a proximal surface binder (DsrA), a distal surface binder (A_18_) or both simultaneously. The complexes were then visualized by native gel electrophoresis (Fig. 3B). As shown in Fig. 3B, F220•H was not disrupted by addition of DsrA or A_18_ alone. A faster migrating species was observed on these gels with increasing concentration of competing RNAs. These complexes may result from conformational changes of the H•F220 complex when a competitor displaces F220 from one face of Hfq. To displace *fhlA*220 from Hfq entirely, both A_18_ and DsrA had to be present. This experiment clearly shows that *fhlA*220 mRNA interacts with Hfq using both the proximal and distal surfaces. Although such a binding mode with Hfq has not been reported, this sort of behavior was previously predicted based on mutagenesis data [28]. In contrast, *fhlA*53 is readily displaced by addition of DsrA (Data not shown). Thus this minimal construct mimics only part of the native interaction with Hfq.

### Secondary structure analysis of *fhlA*220 mRNA

**Figure 4:**
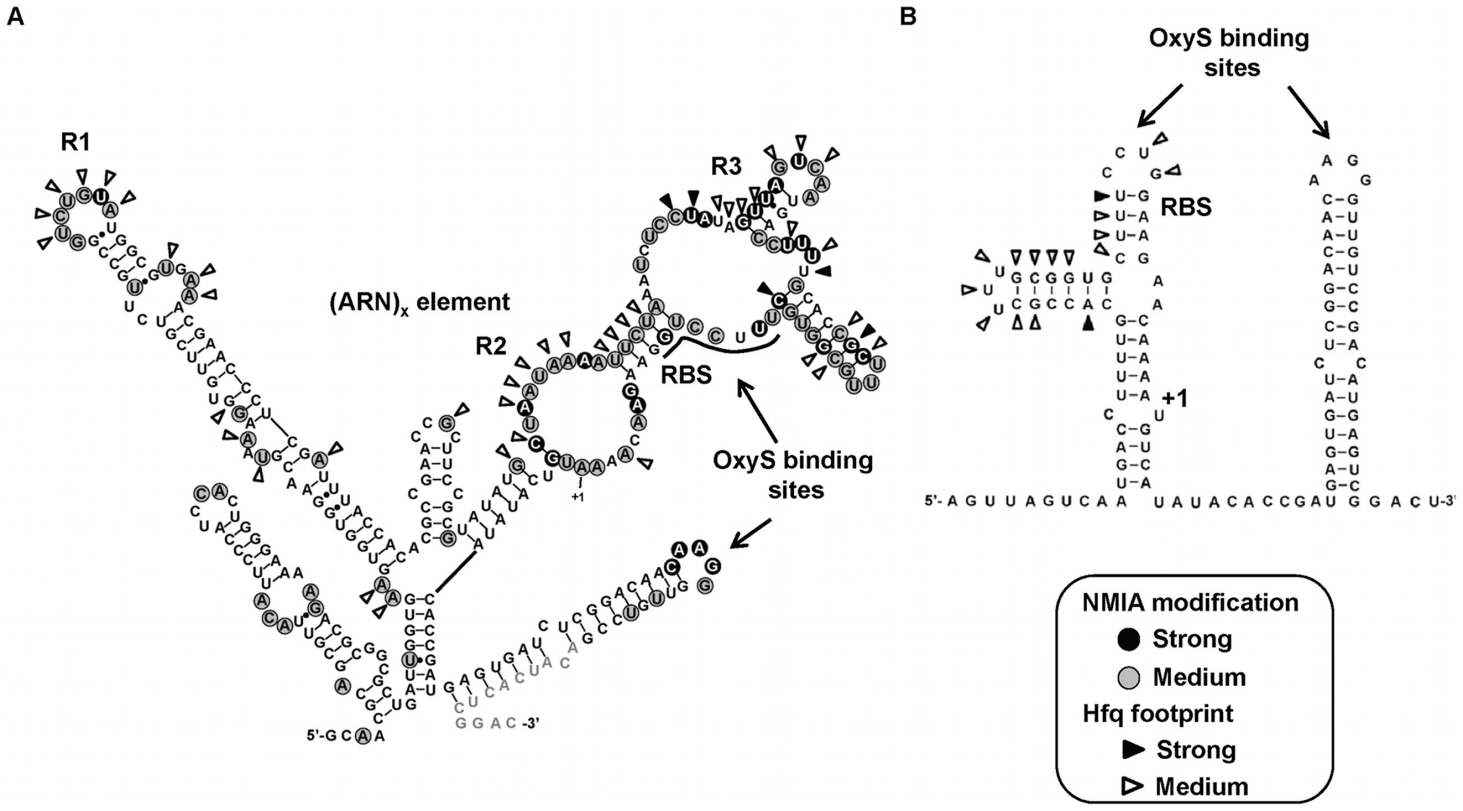
Proposed secondary structure models for *fhlA*220 and *fhlA*53. (A) SHAPE-derived secondary structure model for *fhlA*220 mRNA. The NMIA reactivities are depicted on each base position of the proposed structure. SHAPE reactivities above 0.7 are depicted by dark circles; reactivities 0.2 - to 0.7 are shown in gray circles and reactivities below 0.2 are un-circled. Bases shown in gray text annotations were not analyzed. Hfq footprinting data measured by SHAPE in the presence and absence of 1 µM Hfq are superimposed on the structure model for *fhlA*220. Closed wedges are base positions with strong Hfq footprints, where the relative reactivity was >0.7 and open wedges represent relative reactivities between 0.3 and 0.7. OxyS binding sites, RBS and the (ARN)_x_ elements are also shown. (B) Secondary structure for the previously characterized *fhlA*53. To identify Hfq binding sites of *fhlA*53, terbium mediated footprinting was performed. Strong Hfq footprints at base positions are shown by closed wedges and medium reactivities with Hfq are shown by open wedges.

SHAPE [29], [30] was carried out on *fhlA*220 mRNA to investigate structural and functional elements that might be important for RNP complex formation with Hfq and OxyS. Fig. 4A, shows the experimental secondary structure for *fhlA*220 mRNA derived from SHAPE constraints, superimposed with the modification intensity data. In Fig. 4B, the previously published secondary structure for *fhlA*53 derived from nuclease digestion data is shown for comparison [21]. *fhlA*220 exhibits highly structured regions at both the 5′-region and 3′ ends of the construct and a rather flexible region between positions −76 to +3. It is apparent that *fhlA*220 has distinct functional elements beyond the limits of the previously characterized *fhlA*53 construct.

The two structures differ significantly between −53 and +60. The distinctions in this region arise mainly from bases upstream of −53 pairing with downstream sequences in *fhlA*220. The binding context of *fhlA*53 to OxyS was through two kissing complexes at the leader and coding regions of the mRNA. Only one of the two-stem loop structures remains in the context of *fhlA*220. The proposed stem loop within the upstream region of *fhlA*53 is a bulge in *fhlA*220 and thus remains accessible for OxyS binding. Furthermore the start site and the Shine Dalgano (SD) sequence are unhindered within the flexible region of *fhlA*220. Although the spatial requirement of OxyS to interact with the bulge and the kissing loop seems to be met, complex formation may be hindered by the tertiary structure of *fhlA*220. The 15-fold lower affinity between OxyS and *fhlA*220 relative to *fhlA*53 may be attributed to this structural complexity in the leader sequence and presumably the need to break tertiary contacts to accommodate the bimolecular interaction.

### Hfq binding sites of *fhlA* mRNA

Two methods were used to determine the Hfq binding sites on the leader sequences of *fhlA* - terbium-mediated hydrolysis (*fhlA*53) and NMIA modification (*fhlA*220). Changes in reactivity at each site were categorized as strong or medium and are depicted in Fig. 4, superimposed on the experimental secondary structures (also see Figure S3). Three main regions showed differential activity in the presence and absence of Hfq (labeled R1, R2 and R3 in Fig. 4A). R2 contains a canonical (ARN)_x_ motif like the one identified in *rpoS* mRNA and recently shown to bind the distal face of Hfq [23], [26]. This site was predicted based on sequence analysis and is now a confirmed interaction site. R1 is comprised of a 7nt loop and an adjacent bulge. R3 lies within a highly flexible region adjacent to the upstream portion of the OxyS interaction sequence. Since the competition gel shift assay showed that *fhlA*220 interacts with both distal and proximal binding sites in Hfq, either R1 and or R3 might interact with the proximal face of Hfq while R2 interacts at the distal site.

### Kinetics of *fhlA* association with Hfq

**Figure 5:**
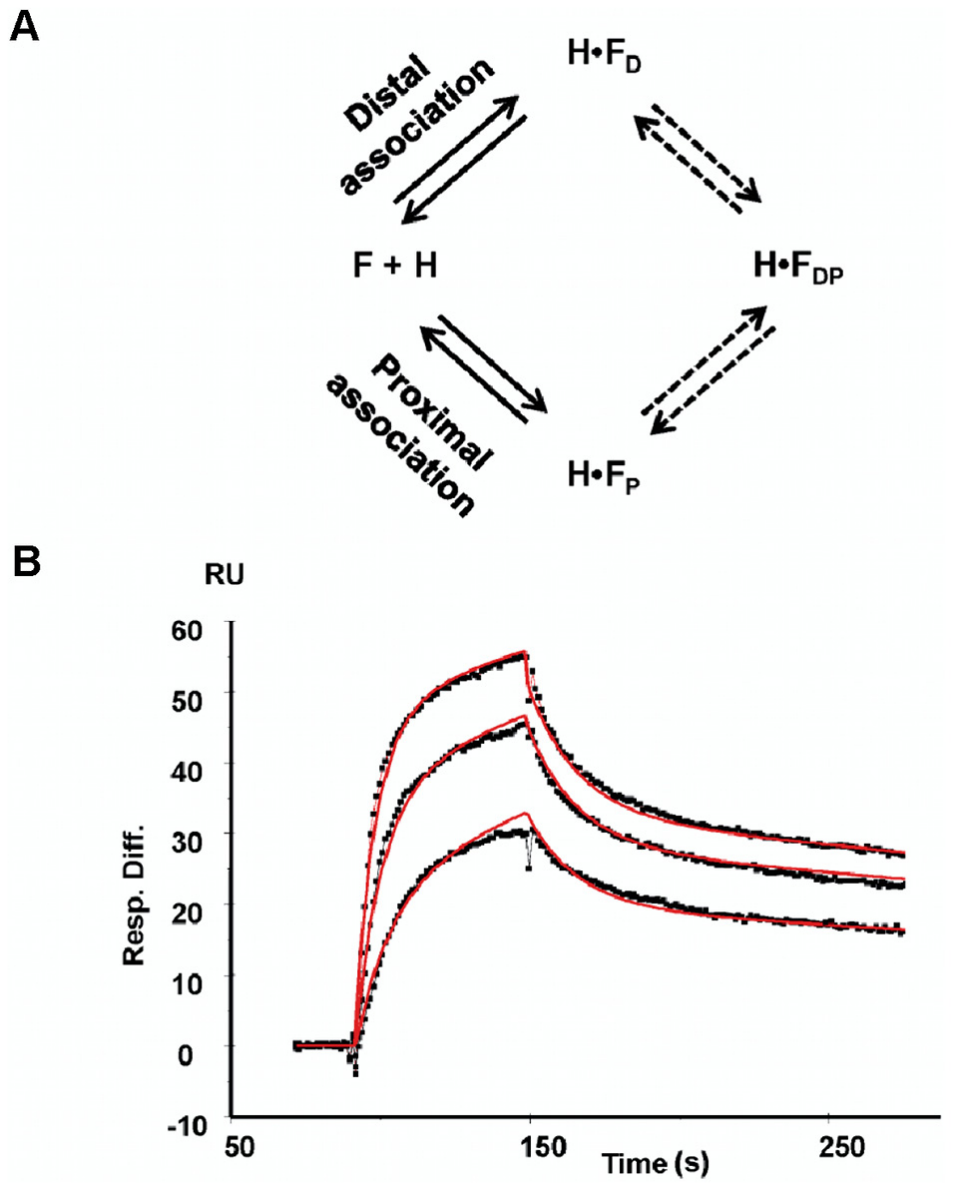
Kinetics of *fhlA* association to Hfq measured using Surface plasmon resonance. (A) The interacting model of Hfq to *fhlA* mRNA. Hfq interaction with *fhlA*220 was viewed as a parallel binding model since Hfq presents two distinct RNA binding surfaces (distal and proximal). The SPR data was fitted into a model that identifies two complex formations through 2-independent pathways reflecting RNA association to distal and proximal sites (line arrows). Formation of the closed complex (dashed arrows) leads to no significant change in the SPR response and was not used in data fitting. (B) Sensorgram for *fhlA*220 interacting with Hfq. Here varying concentrations of Hfq (5–15 nM) were titrated to surface immobilized *fhlA* mRNA. At the end of each injection (Hfq), the dissociation was monitored by flowing buffer over the sensor surface, allowing spontaneous dissociation in the absence of competitors. Solid lines represent the above-mentioned fitting models applied to the data sets (materials and methods).

Kinetic analysis of the binding of Hfq to *fhlA*53 and *fhlA*220 was performed using Surface Plasmon Resonance (SPR). 5′-biotinylated mRNAs were bound to streptavidin coated sensor chips. Hfq was then allowed to bind under various conditions while monitoring the interaction. *fhlA*220 was already shown to interact with both the proximal and distal RNA binding surfaces of Hfq [11], [26], [28]. The kinetic model that was used to fit the binding data for H•F220 is shown in Fig. 5A, providing rate constants that correspond to proximal and distal surface interactions. Since, *fhlA*220 wraps around Hfq in order to interact with both surfaces, one could envision more complex models than that shown in Fig. 5A, but this minimal model was sufficient to fit the data to obtain the magnitudes of the rates in which *fhlA* binds to Hfq.

An example kinetic trace fit to this model is shown in Fig. 5B. Both open complexes (H•F_D_ and H•F_P_) then converge to a single closed H•F_DP_ complex in which *fhlA*220 binds both surfaces of Hfq. This latter step has not been included in the data fitting shown in Fig. 5. The SPR experiment does not appear to be particularly sensitive to this conformational change as it is an internal rearrangement with little change in refractive index in the interfacial zone. Rate data for Hfq interacting with *fhlA*220 and *fhlA*53 are collected in Table 1. Thermodynamic dissociation constants (K_D_s) were computed from kinetic data providing values similar to those measured by gel shift assays for *fhlA*220 (Table 1).

**Table 1:** Kinetics and affinities for *fhlA* constructs associating to wt-Hfq, Y25A Hfq and K56A Hfq measured using the kinetic model shown in Fig. 5A.

	k_a1_ (M^−1^ s^−1^)	k_d1_ (s^−1^)	K_D1_ (nM)	k_a2_ (M^−1^ s^−1^)	k_d2_ (s^−1^)	K_D2_ (nM)	K_D,agg_ (nM)
Hfq-*fhlA*220	(1.60±0.06)×10^6^	(1.40±0.02)×10^−3^	0.83±0.04	(4±1)×10^6^	(5.6±0.8)×10^−2^	13±4	10±2
Hfq-*fhlA*53	(2.0±0.2)×10^6^	(1.0±0.1)×10^−1^	50±10	(3.7±0.7)×10^5^	(1.9±0.2)×10^−3^	5±1	43±6
K56AHfq-*fhlA*220	(2±2)×10^5^	(6.6±0.3)×10^−4^	3±2	(2±1)×10^5^	(7±1)×10^−2^	400±300	131±60
Y25AHfq-*fhlA*220	(2±1)×10^6^	(3.0±0.1)×10^−1^	160±90	(2.9±0.8)×10^5^	(1.0±0.2)×10^−3^	4±1	176±100

K_D,agg_ is the equilibrium constant based on the compound association and dissociation rate constants defined in eq. 6.

Looking at these data, one can draw several significant conclusions regarding the interactions between *fhlA* and Hfq. Two low-nanomolar K_D_s result from fitting to this model and one can also calculate an aggregate dissociation constant (K_D,agg_) of 10±2 nM for this interaction since the observed rates are a combination of the microscopic rate constants associated with distal and proximal site binding. This aggregate value was in good agreement with data obtained using gel shift assays. In contrast, for *fhlA*53 binding to Hfq, only one of the binding interactions produced a K_D_ comparable to that observed with *fhlA*220. K_D,agg_ of 43±6 nM was calculated for this interaction, once again in agreement with gel shift data. This phenomenon likely reflects the loss of the specific interaction with the distal surface due to the lack of the (ARN)_x_ motif.

### OxyS binding kinetics to *fhlA*220 and *fhlA*53

Surface plasmon resonance was also used to measure OxyS binding to *fhlA*220 and *fhlA*53 in the absence of Hfq (Figure S4). 5′-Biotin labeled *fhlA*53 was immobilized and OxyS was titrated at various concentrations. Data were fit to a Langmuir binding model to obtain kinetic parameters. The association rate constant for OxyS interacting with *fhlA*53 was (1.3±0.5)×10^4^ M^−1^s^−1^ while the dissociation rate constant was (6.75±0.08)×10^−4^ s^−1^, yielding a dissociation constant (K_D_) of 50±20 nM. This value is in good agreement with equilibrium data obtained using gel shift assays. To obtain kinetic data for *fhlA*220, the experiment had to be inverted such that 5′-biotin labeled OxyS was immobilized into the SPR sensor surface with subsequent addition of *fhlA*220. As predicted by gel shift assays (Fig. 2B), the OxyS•F220 complex formed weakly with a K_D_ of ∼1.96±0.01 µM estimated from the SPR kinetic constants. This K_D_ is ∼5 fold weaker than that measured using gel shift assays. The weak affinity resulted from slow association, ((9.7±0.4)×10^3^ M^−1^s^−1^), likely due to the need for *fhlA*220 to rearrange, to make the interaction site accessible. These data imply that in the absence of Hfq, binary complexes still form but relatively slowly in both cases and with significantly lower affinity.

### *fhlA*220 interaction with proximal and distal mutants of Hfq

**Figure 6:**
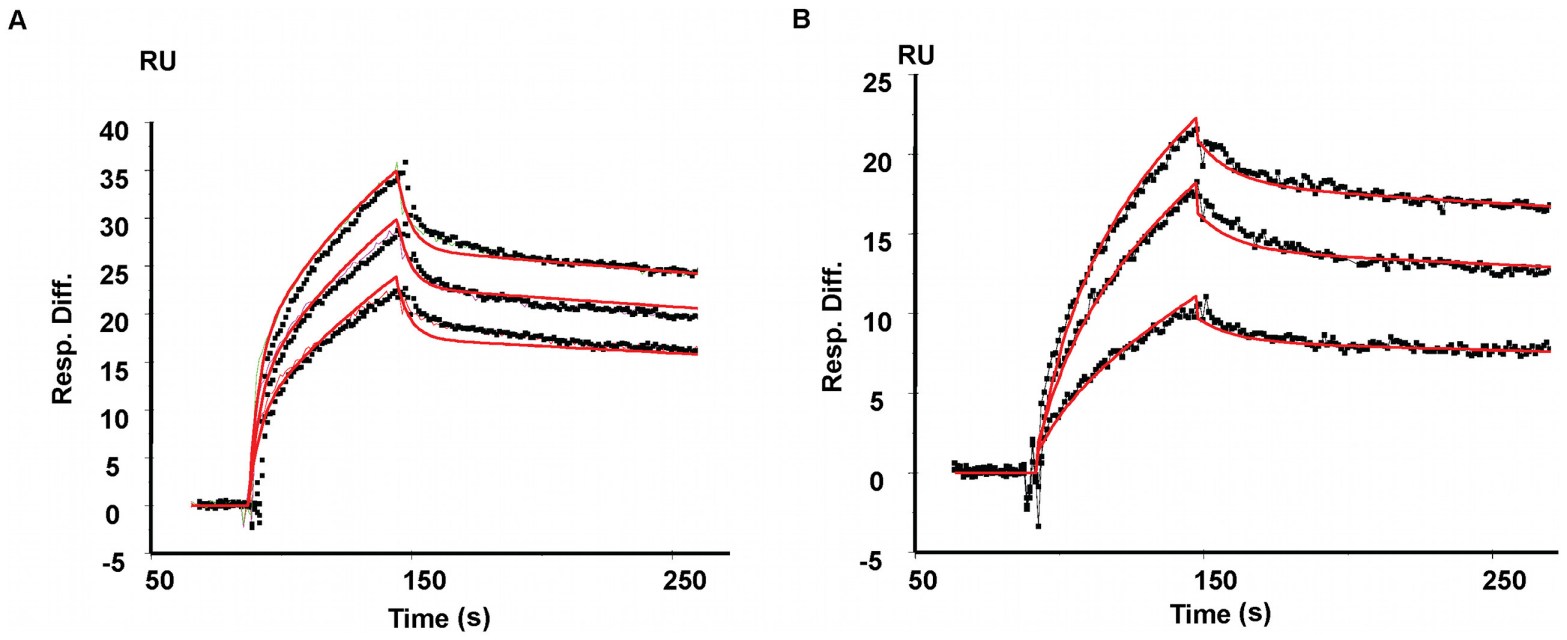
Kinetics of *fhlA*220 mRNA interaction with Y25A and K56A Hfq mutants. (A) Sensorgram for *fhlA*220 binding to Y25A Hfq. It was shown that Y25A mutation abrogated RNA binding at the distal site of Hfq [28]. As described previously ∼3 fmol of mRNA was immobilized in the SPR sensor surface and titrated with mutant Hfq at varying concentrations of 33 nM - to 67 nM hexamer. (B) Sensorgram for *fhlA*220 binding to K56A Hfq. K56A Hfq mutant has been shown to destabilized RNA binding at the proximal surface of Hfq. Hfq was titrated at concentrations of 17 nM - to 50 nM K56A Hfq hexamer. For both (A) and (B), the above-mentioned two-site parallel binding model was applied.

To measure effects of the kinetics when one of the two RNA binding surfaces of Hfq were abrogated, proximal and distal mutants were used. Hfq mutants, Y25A and K56A were previously shown to disrupt RNA interactions at distal and proximal sites of Hfq respectively [28]. Hfq mutants were allowed to interact with *fhlA*220 and kinetic parameters were measured using SPR (Fig. 6 and Table 1). For both Y25A and K56A Hfq, one of the two apparent affinities was dramatically altered while the other was essentially unchanged. K_D,agg_s of 131±60 nM and 176±100 nM were estimated from kinetic data for Y25A and K56A Hfq mutants respectively. This destabilization can clearly be attributed to the partial loss of activity in Hfq caused by mutations essential for RNA binding. Also note that, the overall association rates for *fhlA*220 were diminished for both Hfq mutants compared to wt-Hfq, indicating that Hfq facilitates faster association rates, allowing it to capture the mRNA more effectively by using both RNA binding faces.

### Competing Hfq from the *fhlA*220•Hfq complex using distal and proximal binding RNAs

In the previous section, SPR was used to study the direct dissociation of Hfq from *fhlA*220. Within a cell, a more common scenario might be an exchange reaction where Hfq passes from one RNA to another without ever being free in solution. To measure exchange rates, the H•F220 complex was formed and immediately titrated with DsrA, A_18_ or a mixture of DsrA and A_18_. DsrA is a sRNA associated with cold shock rather than oxidative stress, and binds to the proximal site of Hfq while A_18_ represents a distal site binding RNA [28]. SPR sensorgrams for these experiments are shown in Figure S5. In these experiments, 100 nM Hfq was injected to ∼3 fmol of surface bound *fhlA*220. Once the injection was complete, DsrA and A_18_ (500 nM and 300 nM respectively) were introduced. The dissociation data were fit to a Langmuir dissociation model. Hfq dissociates with a rate constant of 0.07±0.01 s^−1^ when competed with both proximal and distal RNAs, a rate up to 50-fold faster than simple dissociation (Table 1). Addition of A_18_ alone gave practically the same dissociation rate as the mixture (0.06±0.01 s^−1^) whereas the addition of DsrA alone competed for Hfq at a rate 3-fold slower (0.020±0.005 s^−1^) (Figure S5). Both types of competition, however, are faster than the direct dissociation. These results are consistent with the wrap-around model for Hfq binding to mRNAs presented in Fig. 5A. The data are also consistent with an exchange mechanism when 2 RNAs compete for Hfq, but further experiments will be required to validate that such a process really occurs.

## Discussion

Almost all trans-acting sRNAs in *E. coli* and *Salmonella* require Hfq for their gene regulatory activity. In addition to its function of promoting base pairing of sRNAs to their target mRNAs, Hfq is also thought to engage ribosomes, poly A polymerase and RNase E and other enzymes that are involved in RNA transactions [11]. Early models that investigated functional mechanisms for Hfq were sRNA centric however, where the Hfq-sRNA complex sought their mRNA targets within the cell. This hypothesis led most studies to use mRNA constructs that only included structural elements necessary for sRNA binding [21], [22], [31], [32]. These mRNA constructs may have lacked important upstream regulatory sequence elements such as the recently identified (ARN)_x_, motifs that are essential for effective Hfq-mediated gene regulation [23], [25], [33].

One interesting consequence of extending the *fhlA* mRNA leader region was the ability of the mRNA to interact simultaneously with both RNA binding surfaces of Hfq. In the absence of the upstream portion of the leader sequence, *fhlA*53 interacts only with the proximal surface of Hfq. The more complex binding interaction of *fhlA*220 supports a model wherein mRNAs interact with Hfq even in the absence of regulatory sRNAs. This complex can essentially act as an Hfq tag. It can alter basal translation levels alone, while also marking the mRNA as one susceptible to regulation by an sRNA if and when the appropriate sRNA is transcribed. Such a model would enable the cell to respond to stress signals more readily since it streamlines the search for appropriate messages.

The SHAPE-derived secondary structure model proposed for *fhlA*220 provides insight into functional elements in *fhlA*220 mRNA. A region with weak base pairing contains most of the regulatory elements such as the OxyS binding site, RBS, translation start site and the putative (ARN)_x_ motif. This relatively floppy section is flanked on both 3′ and 5′ ends with highly structured regions. When these structures were inadvertently disrupted, such as in the *fhlA*136 species, the ability to bind Hfq was lost indicating the importance of these folds for presenting the Hfq binding motif. Since both RNA binding surfaces of Hfq interact with the *fhlA* leader, multiple footprints were expected. One point of contact was the (ARN)_x_ sequence element, which should interact with the distal surface of Hfq [26]. This leaves regions R1 and R3 as potential proximal binding sites. The A/U rich nature of R3 and its proximity to the (ARN)_x_ motif lead us to propose that this site is more likely than R1 to be the natural proximal binding element in *fhlA*220.

The presence of the Hfq binding motif (ARN)_x_ in the leader sequence was interesting. These A-rich stretches are now widely accepted as being present in mRNA leader sequences that are regulated by Hfq [23], [34]. In a recent study in *Salmonella*, it was projected that Hfq modulated the synthesis of ∼20% of all proteins either directly or indirectly [19]. A similar number was predicted by a brief bioinformatic survey of potential Hfq binding regions (AAYAA and ARN tracts) in upstream sequences within *E. coli* mRNAs [26]. These sequence elements are quite degenerate and thus will appear with a high frequency by chance. Whether all of these AAYAA and (ARN)_x_ elements represent Hfq binding sites requires further investigations as it is possible that an additional structural context is required to define an Hfq-dependent regulatory element within an mRNA.

Elongation of the *fhlA* leader from −53 to −220 significantly alters both the kinetics and thermodynamics of its interactions with Hfq and OxyS. Complex formation with OxyS was much weaker for *fhlA*220 relative to *fhlA*53 in the absence of Hfq. Addition of Hfq, however reversed this trend and restored OxyS affinity for the Hfq•*fhlA*220 complex. This finding implies the requirement of Hfq to facilitate regulatory RNP complex formation as observed in in vivo assays for this system [35]. Similar observations were made by Soper *et al.* in the *rpoS* leader interaction with Hfq. Hfq binds more tightly to *fhlA*220 than to *fhlA*53. This extra stabilization results from the upstream binding element that enables *fhlA*220 mRNA to interact Hfq through both the proximal and distal surfaces simultaneously.

The kinetic model used to characterize *fhlA* binding to Hfq measured two compound rate constants. By using distal (Y25A) and proximal (K56A) face mutants, a large overall destabilization in affinities were observed for *fhlA* binding to Hfq. This result indicated that both binding surfaces of Hfq were in use and validated the competition gel shift experiments. Association of *fhlA*220 to Hfq was rapid, with similar magnitudes observed for both rate constants. The shorter *fhlA*53 construct on the other hand showed very different binding behavior with a 50-fold difference in the dissociation rates between the two apparent rates, explaining the propensity for *fhlA53* to binding to a single site (proximal).

Simple dissociation may not represent the natural behavior of Hfq in the cell however. With so much Hfq and so many Hfq-binding RNAs, an exchange from one RNA to another may be a more common behavior. We therefore exposed the Hfq•*fhlA*220 complex to other RNAs to determine their ability to induce exchange. This experiment showed faster exchange rates than were observed for simple dissociation with the most pronounced effect occurring when a distal binding RNA was introduced. The competing RNA essentially traps one of the two Hfq binding sites facilitating complete dissociation of *fhlA220* from Hfq. This finding has functional importance since most sRNAs bind through the proximal site. Thus, if Hfq is bound to both proximal and distal sites of an mRNA in a closed complex, exposure to a sRNA will lead to formation of a ternary complex that retains Hfq contact with the mRNA through the (ARN)_x_ motif and Hfq's distal surface. If complementarity between the RNAs is found within the lifetime of the ternary complex, the conformational changes responsible for gene regulation will ensue. Otherwise, the sRNA will simply dissociate leaving the Hfq-mRNA complex intact and unchanged. Hfq-RNA complexes challenged with the distal-binding A_18_ RNA, showed a three-fold kinetic advantage over dissociation with proximal binding DsrA. These findings imply that the Hfq-mRNA complex will be more resistant to a non-cognate sRNA than to an A-rich RNA that resembles an alternative mRNA. Whether these findings are consistent with other Hfq binding mRNAs besides *fhlA* remains to be validated.

In conclusion the data presented here supports the notion that Hfq-mRNA complexes are essential elements in sRNA mediated gene regulation. This work supports recent findings by the Woodson lab that sequence elements in upstream regions of mRNAs are important for Hfq binding and gene regulation in vivo [33]. These (ARN)_x_ motifs are widely dispersed in bacterial mRNAs and we are only now learning about the importance of such signals in bacterial genes. A recent report suggested an Hfq interaction with the RNA polymerase β-subunit [36] and might imply the potential to handoff of Hfq to nascent transcript marking them for subsequent regulation by an sRNA if necessary in response to an environmental signal. This would be an efficient way for Hfq to locate target mRNAs and ensure that they are positioned properly to support sRNA-mediated gene regulation if required.

## Materials and Methods

### Plasmid construction for *fhlA*53 and OxyS

pNS10901 carries the *fhlA* 5′-end fragment from −53 upstream of the AUG initiation codon to +60. The construct was prepared by positioning the dsDNA of the FhlA fragment behind a T7 promoter sequence flanked by a EcoR I and a BamH I site using the following two primers 5′-ACGTACGAATTCTAATACGACTCACTATAGGCAGTTAGTCAATGACCTTTTGCACCGCTTTGCGGTGCTTTCCTGGAAGAAC-3′ and 5′-CGAGCTGGATCCAATATTTGTTGTCCGAGTGATGTCGAACAACCCTTGTTGTCCGAGA TCACTCATCGGTGCATATGACATTTTGTTCTTCCAGGAAAGCACCGC-3′. The primer extension assay was performed using a standard procedure described previously [37]. The resulting DNA was cloned into pUC19 and used to transform into XL-10 supercompetent *E. coli* cells. The resulting plasmid (pNS10901) was verified by sequencing. The plasmids (pNS10901) were isolated using a Giga-prep kit (Qiagen). Plasmid DNA were further purified using phenol-chloroform-isoamyl extraction and ethanol precipitated. The plasmid was prepared for runoff transcription by digesting with Ssp I. The OxyS fragment was amplified in XL-10 *E. coli* cells using primers OxyS F- 5′-GGAACAAGCTTTAATACGACTCACTATACCTTCGCCTAGGACCTCTAGG-3′ and OxyS-R- 5′-CCGAGCGAATTCTTTAAAGCCTCGCCGTGGAG-3′.

The primers were designed with a T7 RNA promoter in the OxyS-F primer with a flaking EcoR I and Hind III restriction sites. The dsDNA fragments were inserted into pUC19 and transformed into XL-10 *E. coli* cells (pNS10092). The plasmids were verified using sequencing. The plasmid was prepared for runoff transcription by digesting using Dra I.

### RNA preparation for SHAPE, in vitro binding and kinetic analysis

A_18_ RNA was purchased from Dharmacon Technologies and deprotected following the manufacturer's protocol. RNA quality was assessed using denaturing PAGE and gel purified. For in vitro binding and kinetic analysis, *fhlA*220 mRNA were transcribed using a DNA fragment that amplifies the FhlA gene from −220 to +60 in *E. coli* XL-10 cells using primers 5′-GGAACCGAATTCTAATACGACTCACTATAGCAGCGTTACATTCCCATCCACTGG-3′ and 5′-CCGAGCGGATCCAATATTTGTCCGAGTGATGTCGAACAACCC-3′ and digested with Ssp I before transcribing. For SHAPE analysis, *fhlA*220 mRNA was in vitro transcribed using a DNA template that was amplified in XL-10 cells using primers 5′-GGAACCGAATTCTAATACGACTCACTATAGGCCTTCGGGCCAAGCAGCGTTACATTCCCATCCACTG-3′ and 5′-CCGAGCGGATCCAATATTGAACCGGACCGAAGCCCGATTTGGATC CGGCGAACCGGATCGATGTCCGAGTGATGTCGAACAACCC-3′ that includes a structure cassette in the 5′ and 3′ regions as previously described [29]. In vitro transcription was performed after digesting the amplified product with Ssp I. DsrA was obtained by runoff transcription of pBAU10301 that was digested by Ssp I [38].

### Biotinylation of RNA

mRNAs *fhlA*220 and *fhlA*53 was 5′-labled with biotin to be used in surface plasmon resonance experiments. RNAs were first treated with Calf Intestinal Phosphatase (CIP) and phosphorylated using ATP-γ-S using the Ambion Kinase Max kit (Ambion, Inc). In brief, 1 nmol of RNA was treated with CIP (in 10× dephosphorylation buffer, 0.5 units of CIP at 37°C for 2 h). The reaction mixture was purified using the Phosphatase Removal Reagent as described by the product manual. Purified RNAs were phosphorylated with ATP-γ-S using T4 Polynucleotide kinase. Phosphorylated RNAs were purified using a G-25 spin column (GE Healthcare) and speed vacuumed to dryness. RNAs were then dissolved in 45 µl of 100 mM KHPO_4_, pH 8.0, 5 µl of 20 mM N-iodoacetyl-N-biotinylhexylenediamine dissolved in DMF (EZ-Link Iodoacetyl-LC-Biotin, Thermo Scientific). The reaction was incubated at 45°C for 1 h while shaking under dark conditions. The reaction was then ethanol precipitated and analyzed using PAGE.

### Hfq expression and purification

Expression and purification of Hfq and its mutants Y25A and K56A was performed as previously described [28].

### Electrophoretic mobility shift assays

All binding reactions were performed in 50 mM Tris-HCl pH 7.5,100 mM KCl and 10 mM MgCl_2_ at room temperature. Prior to any interaction all RNAs in buffer were annealed at 90°C for 3 min, cooled to room temperature for 30 min. For all reactions 8 µl aliquots were loaded after diluting with loading buffer (10% (w/v) sucrose, xylene cyanol, bromophenol blue) under a power of 5 W on native 5–8% polyacrylamide (37∶1) gel in 1× TBE. Dried gels were visualized by phosphorimaging (Molecular Dynamics) using a Typhoon 9210 imaging system (Amersham). Quantification was done using ImageQuant 5.1 (Molecular Dynamics) and Kaleidagraph 3.0 (Synergy). Data were fit using nonlinear least-square analysis to a cooperative binding model shown below (eq. 1). Here, L is the ligand concentration and the cooperatively is indicated by n. Typical values for n ranged from 1.1 to 2.7.
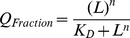
(1)


In the case of A_18_, DsrA competition assays, the *fhlA*220•Hfq complex was pre-formed and A_18_ and DsrA was titrated at varying concentrations from 0 to 30 µM.

### Chemical SHAPE analysis

The secondary structure of *fhlA*220 was mapped using SHAPE chemistry as described previously [29], [39]. In brief 1 pmol of RNA was folded in buffer (50 mM Tris-HCl pH 7.5, 100 mM KCl, 10 mM MgCl_2_) by heating to 95°C and cooled to room temperature for 15 min. Then added N-methylisatoic anhydride (NMIA) in anhydrous DMSO to a final concentration of 3 mM. The reactions were incubated at 37°C for 45 min. A control experiment without NMIA was performed for 1 pmol of RNA where instead of NMIA, DMSO was added. The reaction was then ethanol precipitated in the presence of a co-precipitant (20 µg, Glycogen).

The 2′-O-adducts were analyzed using primer extension. The modified/unmodified RNAs (1 pmol, 10 µl, in 0.5× TE) were heated to 95°C for 3 min in a thin PCR tube and cooled in ice for 1 min. Fluorescently labeled primer (5′-F-GAACCGGACCGAAGCCCG) (3 µl) was added to (+NMIA) (0.3 µM WellRED D4) and (−NMIA) (0.4 µM WellRED D3) reactions respectively. The primer template solutions were then incubated at 65°C for 5 min and 37°C for 15 min. Primer extension was initiated by adding enzyme mix (4 µl of Superscript III FS buffer, 1 µl 0.1 M DTT, 1 µl 10 mM dNTP mix) and incubating at 52°C for 1 min. Then added Superscript III (1 µl) and incubated at 52°C for 15 min. In addition to these two reactions two sequencing reactions were performed to identify corresponding peaks. The sequencing reactions were assembled as mentioned above (RNA, 1 pmol), 3 µl primer (ddCTP, 1.2 µM LICOR IR 800) and (ddATP, 2 µM WellRED D2), 6 µl enzyme mix, ddNTP (1 µl, 0.25 mM) and Superscript IIII (1 µl) and performed RT. The four reactions (+NMIA), (−NMIA) and sequencing reactions were combined and ethanol precipitated in the presence of glycogen. The pellets were washed twice with 70% ethanol and dried under vacuum. The pellets were re-suspended in SLS loading solution (Beckman). cDNA samples were separated on a Beckman CEQ 8000 DNA sequencer. The separation was performed using the following parameters (capillary temp: 60°C, denature temp: 90°C, time 150 s, injection voltage: 2 kV, time 7 s, separation voltage 3 kV and separation time 100 min).

The raw fluorescence intensities were analyzed using the software ShapeFinder [40]. The quantitative shape data were normalized to a scale that falls between 0 for un-reactive sites and reactive bases would attain an average reactivity of 1. This method of normalizing was extensively described elsewhere [41]. SHAPE reactivities were then imported to RNAstructure software where the intensities were converted to pseudo-free energy changes [42].

### Chemical footprinting

To identify Hfq binding sites in *fhlA*220, SHAPE chemistry was performed in the presence and absence of Hfq as described above. 2 pmol of RNA was reacted with 1 µM Hfq for 30 min. The RNA was then modified using NMIA (3 mM) at 37°C for 45 min, and treated with Proteinase K (1 µl, 20 mg/mL) at 37°C for 30 min. The reaction was then phenol-chloroform-isoamyl alcohol extracted followed by ethanol precipitation. The primer extensions were performed as previously described and the (+Hfq) base reactivities were compared to (−Hfq) reactions.

Tb(III) mediated footprinting was performed as previously described [14]. In brief, 250K cpm of 5′ ^32^P-end labeled RNA was incubated in probing buffer (50 mM Tris-HCl at pH 8.0, 100 mM NaCl, 10 mM MgCl_2_) containing 0 and 1 µM Hfq hexamer for 30 min at room temperature. TbCl_3_ was added to a concentration of 100 mM and incubated for 2h. The reaction was quenched by adding EDTA (50 mM) and SDS (0,1%). Samples were then treated with Proteinase K and incubated at 37°C for 30 min. The mixture was then resolved on a 8% denaturing PAGE. The data was then analyzed using SAFA software to normalize, align and measure reactivities of base positions with reference to a ladder [43].

### Surface Plasmon resonance

Kinetic experiments were performed on a Biacore 2000 instrument. Experiments were done on either a streptavidin-coated chip (SA chip, Biacore) or CM5 chip where 3000 RUs (response units) of streptavidin was coated using amine coupling. Streptavidin coating the CM5 chip involved, activating the carboxymethylated dextran (CM) sensor chip with 35 µl of 0.2 M EDC and 0.05 M NHS. A 35 µl solution of streptavidin (200 µg/mL in 10 mM sodium acetate, pH 4.8) was injected repeatedly to achieve the expected 3000 RUs of surface coverage. This was then followed by the injection of 35 µl of 1 M ethanolamine to quench residual NHS esters. The Immobilization was carried out at 25°C at a flow rate of 5 µl/min.

All experiments were performed in the same reaction buffer (50 mM Tris-HCl, pH 7.5, 100 mM KCl and 10 mM MgCl_2_). During all experiments ∼3 fmol of 5′ biotin labeled RNAs were immobilized on the sensor chip. Immobilizations of RNAs were performed at a flow rate of 3 µl/min to make sure homogeneous surface coverage is attained. Experiments were carried out at 25°C and at a flow rate of 30 µl/min.

To measure kinetics of OxyS binding to *fhlA*53, OxyS was titrated at varying concentrations of (400 nM, 200 nM and 100 nM). Kinetics of OxyS interacting with *fhlA*220 was measured by immobilizing ∼3 fmols of 5′-Biotin labeled OxyS and titrating *fhlA*220 at concentrations (1–4.5) µM. Surface regeneration was performed by injecting 300 µl of regeneration buffer (50 mM Tris-HCl pH 7.5, 20 mM EDTA) at a flow rate of 100 µl/min. In case of Hfq binding to *fhlA*220 or *fhlA*53 Hfq was titrated at varying concentrations of (15 nM, 10 nM and 5 nM) hexamer. Hfq for this experiment was dialyzed with the reaction buffer using a Slide-A-Lyzer® mini dialysis kit (Thermo Scientific, 3500 MWCO) prior to the experiment. The regeneration was performed by injecting 60 µl of 500 nM DsrA and A_18_ RNA solution. For Y25A Hfq binding to *fhlA*220 Hfq hexamer concentrations at 67 nM, 50 nM and 33 nM were used. For K56A Hfq concentrations of 50 nM, 33 nM and 17 nM were used.

To measure the exchange kinetics between Hfq and *fhlA*220, the co-injection mode that was available in the Biacore 2000 control software was used. Here 17 nM wt-Hfq was titrated and immediately after the injection, either DsrA, A_18_ or both together at concentrations of 500 nM and 300 nM was introduced.

The data were analyzed globally by fitting both the dissociation and association (where applicable) phases simultaneously (BIA evaluation software version 4.1). A 1∶1 (Langmuir) model (two fitting parameters) and a parallel reaction model (four fitting parameters) were used (Fig. 5A). The binding model was constructed in BIA evaluation software and the equation for the model is shown below, making the assumption that 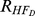
≈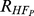 due to similar molecular weights for the two complexes.
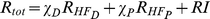
(2)Where,
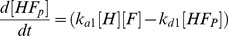
(3)

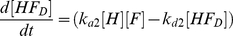
(4)

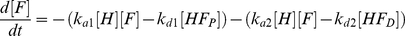
(5)

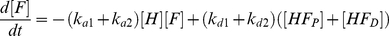
(6)


Total changes in response units for this model was due to contributions from the distal and proximally coordinated complexes (*χ_D_* (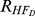) and *χ_P_* (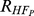), respectively) and *RI*, which corresponds to the bulk refractive index contribution to the overall response (*R_tot_*). Here species *χ_D_* and *χ_P_* represents the mole fractions of the Hfq complexes formed with the distal and the proximal sites respectively. Surface immobilized *fhlA* mRNA is represented as *F* and Hfq is depicted as *H* in above equations. Kinetic rates *k_a1_* and *k_a2_* define association phase parameters whereas dissociation rates are given by *k_d1_* and *k_d2_* for the two binding phases. BIA evaluation uses Marquardt-Lavenberg algorithm to optimize parameters in fits and assigns kinetic constants to the above described equation. The goodness of the fit was judged by the reduced chi-square (χ^2^) values.

## Supporting Information

Figure S1 Quantitative analysis gel shift assays. (A) Analysis of gel shift assays shown in Figure 2B. Binding of F220 to OxyS (closed squares) and ternary complex formation between F220, OxyS and Hfq (closed circles). (B) Quantization of thermodynamic constants for gel shifts for Hfq binding to F53 (closed squares) and F220 (closed circles). As described in materials and methods thermodynamic constants were determined by nonlinear least-square analysis fitted to a cooperative binding model.
(0.93 MB TIF)

Figure S2 *fhlA*220 interaction with Hfq. Gel shift assay wherein [5′-^32^P]- *fhlA*220 mRNA was titrated with increasing concentration of Hfq in the range of 0 to 1.67 µM hexamer.
(0.59 MB TIF)

Figure S3. *fhlA*53 mRNA interaction with Hfq. (A). Gel shift assay for Hfq•*fhlA*220 binary complex formation. (B) Poly-acrylamide gel showing the effect of Hfq binding on Tb(III)-mediated cleavage of ^32^P-*fhlA*53. (C) Quantitative analysis of Hfq binding based on the gel in panel B. Data are represented as a ratio of the intensity of each band in the absence and presence of 1 µM Hfq hexamer. Values greater than 1 represent protection. Data between +1 and −1 were considered to be no significant effect.
(2.28 MB TIF)

Figure S4 Kinetic analysis of OxyS interaction with *fhlA*220 and *fhlA*53. (A) Representative SPR sensorgram for OxyS•*fhlA*53 interaction is shown. 5′-Biotin labeled *fhlA*53 mRNA was immobilized and varying concentrations of OxyS was titrated (400, 200 and 100 nM). (B) SPR sensorgram for OxyS binding to *fhlA*220. Here the biotin label was added to OxyS sRNA and titrated with *fhlA*220 to monitor the interaction (1.5, 3 and 4.5 µM). For both interactions data were fitted into a Langmuir binding model to yield kinetic constants. The model is an over-simplification of a complex system as it ignores unimolecular RNA structural rearrangements that might be required prior to association, but the model sufficient to illustrate the interactions in the absence of Hfq and their approximate rates.
(0.54 MB TIF)

Figure S5 Handoff kinetics of Hfq from Hfq•*fhlA*220 complex. (A) Overview of handoff experiment. The Hfq•*fhlA*220 complex was pre-formed and dissociation kinetics of Hfq were monitored by titrating competing RNAs DsrA (proximal), A_18_ (distal) or both DsrA and A_18_. (B) Sensorgram of Hfq dissociation from the Hfq•*fhlA*220 complex in the presence of 500 nM and 300 nM DsrA and A_18_. (C) Sensorgram of Hfq dissociation from the Hfq•*fhlA*220 complex in the presence of 500 nM and 300 nM DsrA. (D) Sensorgram of Hfq dissociation from the Hfq•*fhlA*220 complex in the presence of 500 nM and 300 nM of A_18_. All dissociation data were fitted in to Langmuir model.
(1.02 MB TIF)
