# Supplementary figures and images for: An Upstream Hfq Binding Site in the fhlA mRNA Leader Region Facilitates the OxyS-fhlA Interaction
Source: PLoS One. 2010 Sep 28;5(9):e13028. doi: 10.1371/journal.pone.0013028 (PMC2946933; doi:10.1371/journal.pone.0013028)

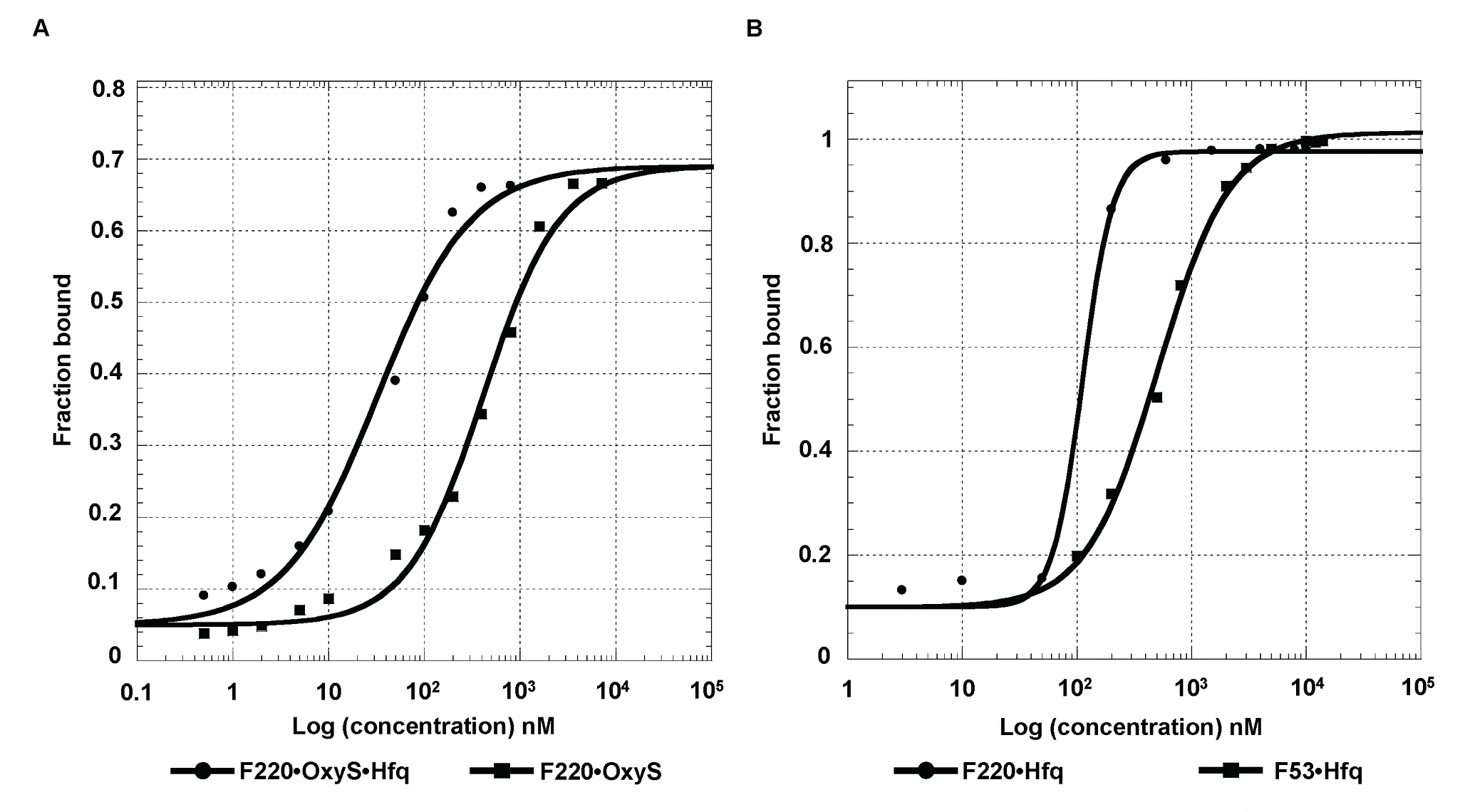

Supplement: Figure S1 — Quantitative analysis gel shift assays. (A) Analysis of gel shift assays shown in Figure 2B. Binding of F220 to OxyS (closed squares) and ternary complex formation between F220, OxyS and Hfq (closed circles). (B) Quantization of thermodynamic constants for gel shifts for Hfq binding to F53 (closed squares) and F220 (closed circles). As described in materials and methods thermodynamic constants were determined by nonlinear least-square analysis fitted to a cooperative binding model. (0.93 MB TIF) [file pone.0013028.s001.tif]

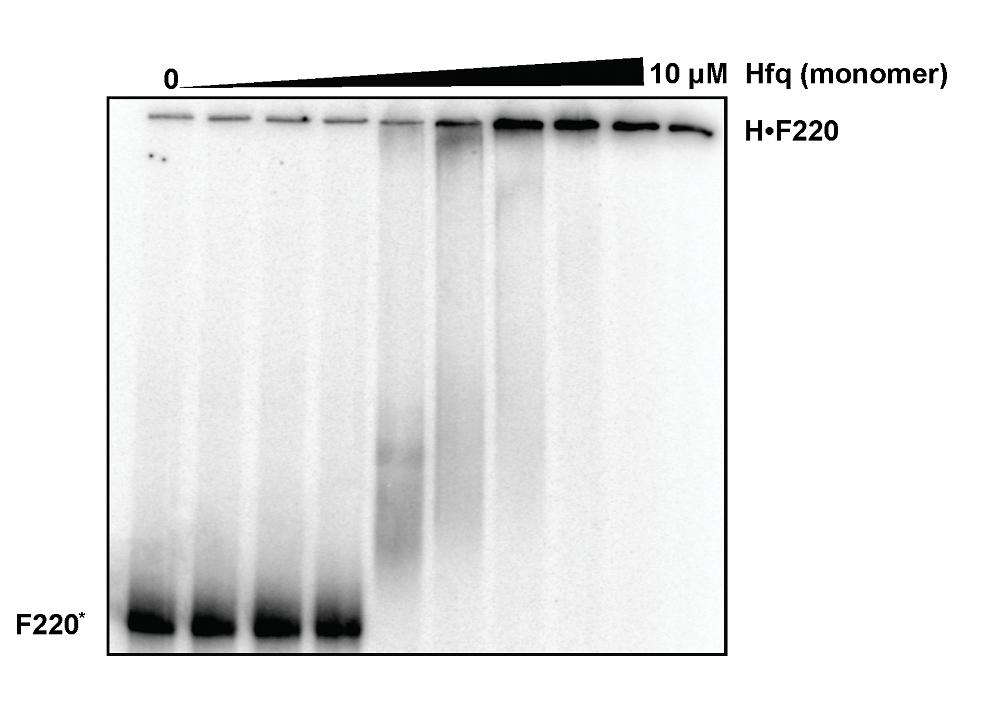

Supplement: Figure S2 — fhlA220 interaction with Hfq. Gel shift assay wherein [5′-32P]- fhlA220 mRNA was titrated with increasing concentration of Hfq in the range of 0 to 1.67 µM hexamer. (0.59 MB TIF) [file pone.0013028.s002.tif]

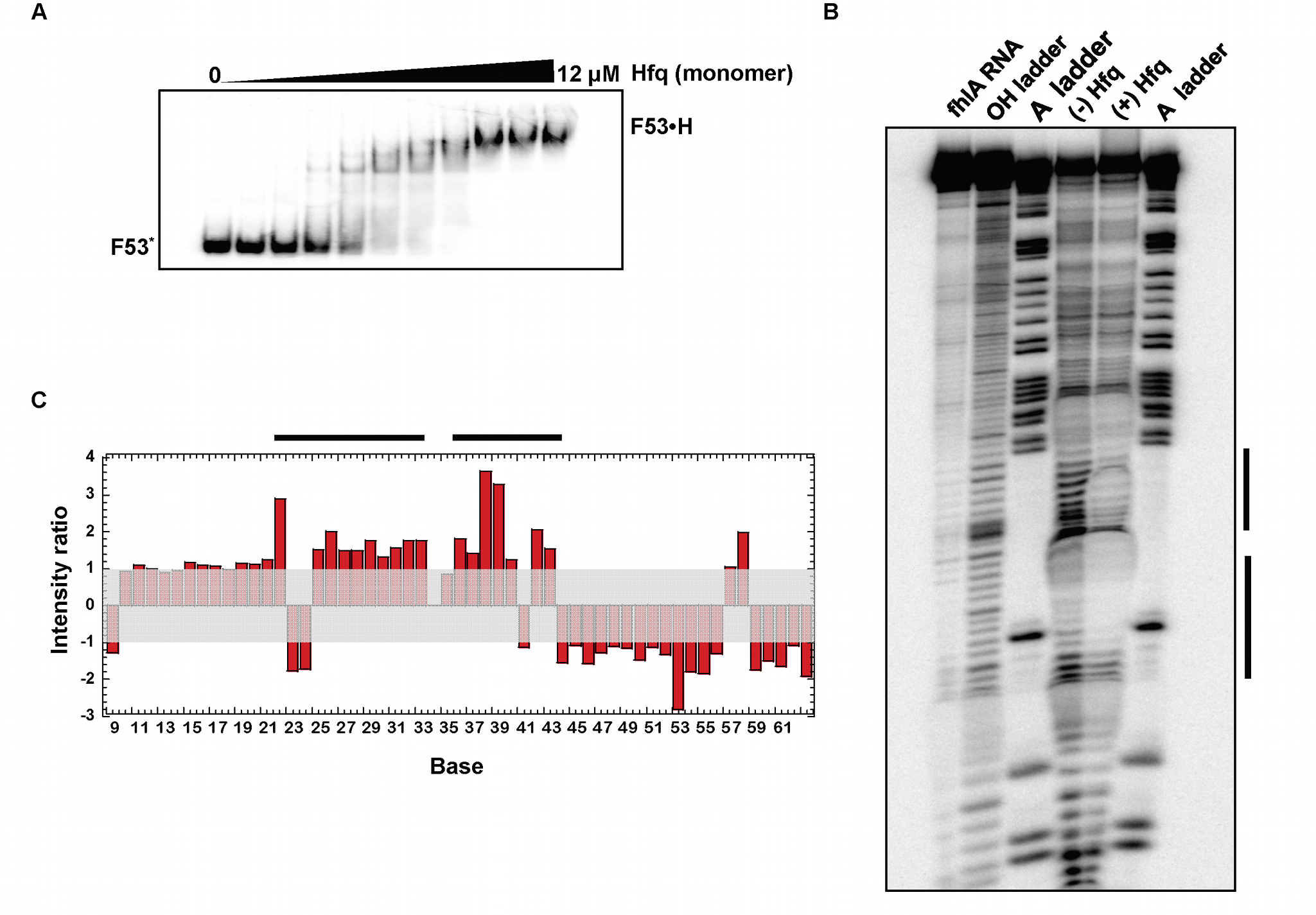

Supplement: Figure S3. — fhlA53 mRNA interaction with Hfq. (A). Gel shift assay for Hfq•fhlA220 binary complex formation. (B) Poly-acrylamide gel showing the effect of Hfq binding on Tb(III)-mediated cleavage of 32P-fhlA53. (C) Quantitative analysis of Hfq binding based on the gel in panel B. Data are represented as a ratio of the intensity of each band in the absence and presence of 1 µM Hfq hexamer. Values greater than 1 represent protection. Data between +1 and −1 were considered to be no significant effect. (2.28 MB TIF) [file pone.0013028.s003.tif]

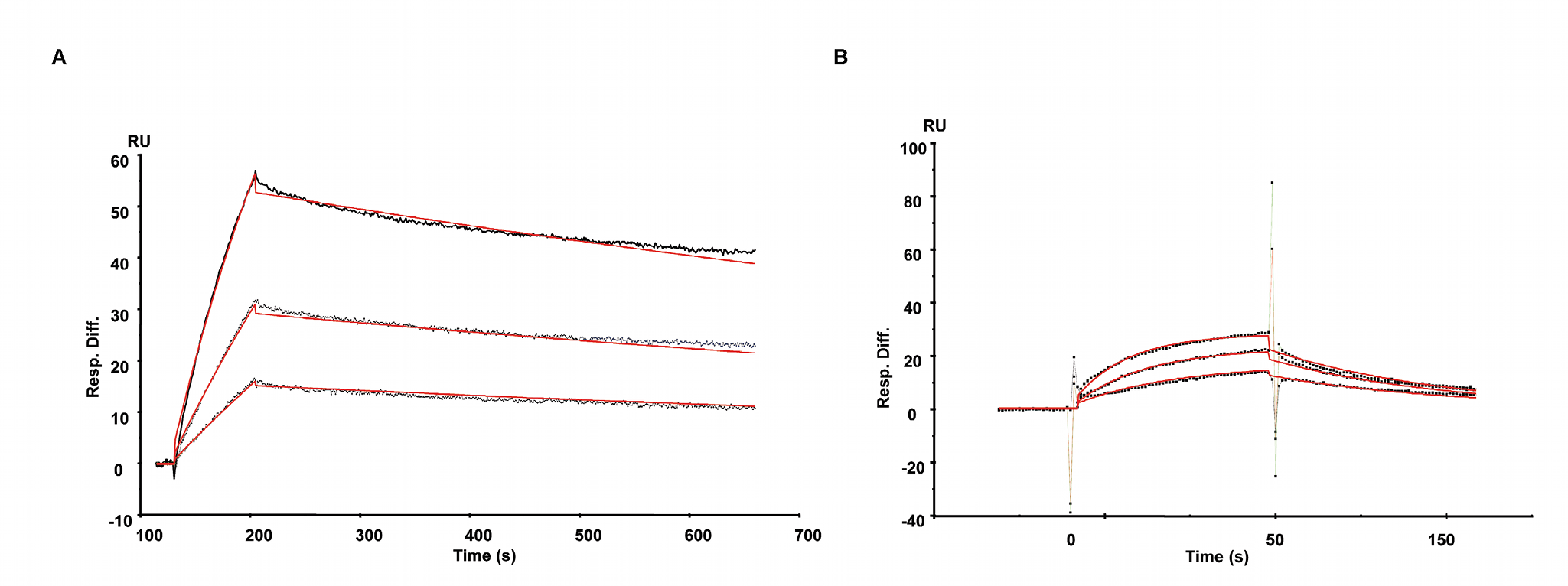

Supplement: Figure S4 — Kinetic analysis of OxyS interaction with fhlA220 and fhlA53. (A) Representative SPR sensorgram for OxyS•fhlA53 interaction is shown. 5′-Biotin labeled fhlA53 mRNA was immobilized and varying concentrations of OxyS was titrated (400, 200 and 100 nM). (B) SPR sensorgram for OxyS binding to fhlA220. Here the biotin label was added to OxyS sRNA and titrated with fhlA220 to monitor the interaction (1.5, 3 and 4.5 µM). For both interactions data were fitted into a Langmuir binding model to yield kinetic constants. The model is an over-simplification of a complex system as it ignores unimolecular RNA structural rearrangements that might be required prior to association, but the model sufficient to illustrate the interactions in the absence of Hfq and their approximate rates. (0.54 MB TIF) [file pone.0013028.s004.tif]

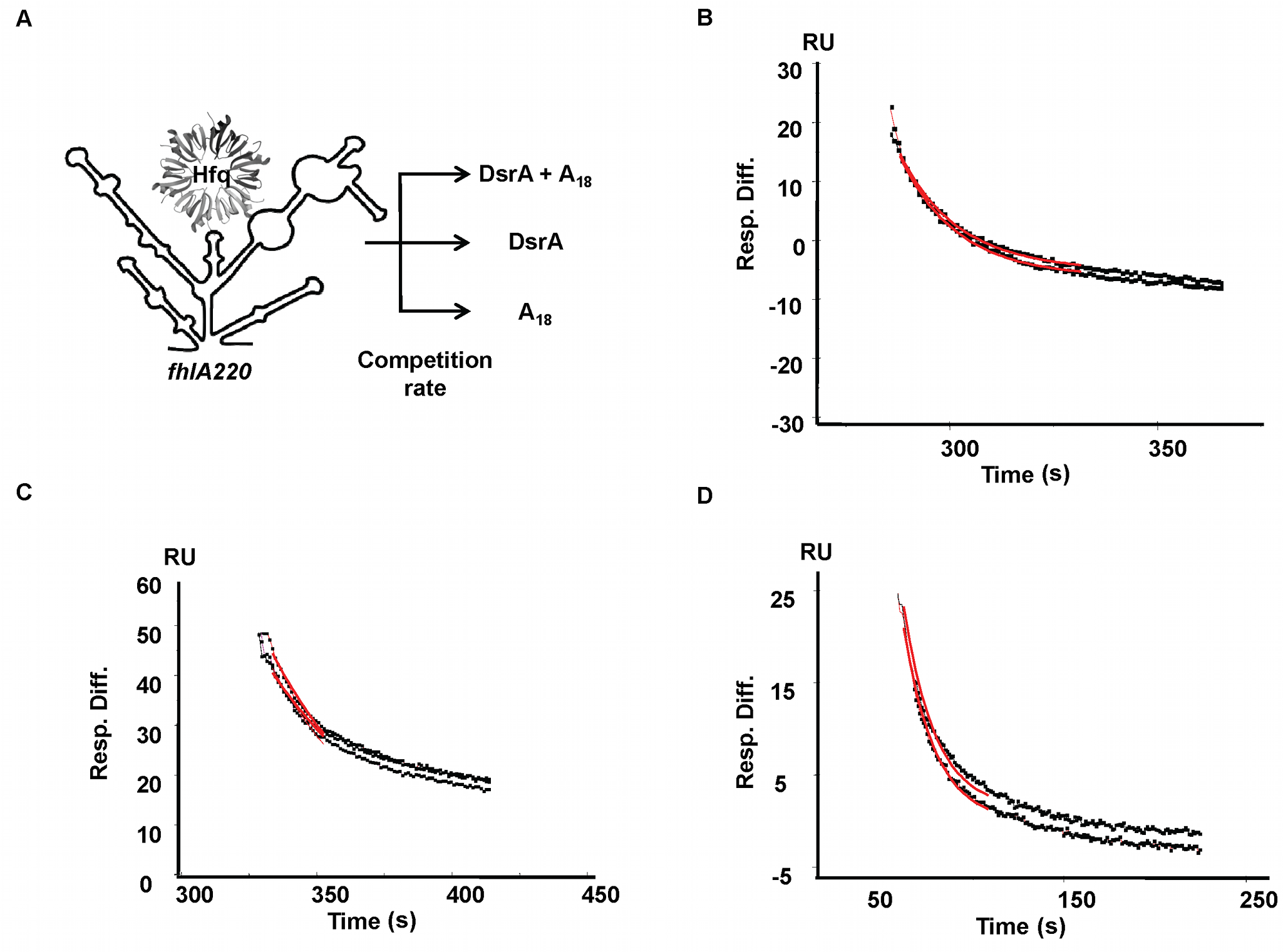

Supplement: Figure S5 — Handoff kinetics of Hfq from Hfq•fhlA220 complex. (A) Overview of handoff experiment. The Hfq•fhlA220 complex was pre-formed and dissociation kinetics of Hfq were monitored by titrating competing RNAs DsrA (proximal), A18 (distal) or both DsrA and A18. (B) Sensorgram of Hfq dissociation from the Hfq•fhlA220 complex in the presence of 500 nM and 300 nM DsrA and A18. (C) Sensorgram of Hfq dissociation from the Hfq•fhlA220 complex in the presence of 500 nM and 300 nM DsrA. (D) Sensorgram of Hfq dissociation from the Hfq•fhlA220 complex in the presence of 500 nM and 300 nM of A18. All dissociation data were fitted in to Langmuir model. (1.02 MB TIF) [file pone.0013028.s005.tif]
